# Changes in Medical Schools

**Published:** 1850-08

**Authors:** 


### THE WESTERN JOURNAL.

THIRD SERIES.—VOL. VI.—NO. II.

LOUISVILLE, AUGUST 1, 1850.

### CHANGES IN MEDICAL SCHOOLS.

Dr. S. H. Dickson has resigned the Chair of the Theory and Practice of Medicine in the University of New York, and returned to the Medical College of South Carolina, in Charleston. He is succeeded by Dr. William Detmold. In the same school other changes have been made. Professor Paine, "who formerly taught the Institutes of Medicine in connection with Materia Medica," has requested to be relieved of the former branch, and in future, in lieu of it, will lecture on Therapeutics, Physiology having been assigned to Dr. Draper, the accomplished Professor of Chemistry. Dr. Paine is undoubtedly a gentleman of great learning, of unsurpassed industry, of unwearying perseverance; but he belongs to the antiquated school of physiologists of which he stood almost the sole specimen and representative. He remained true to his faith, to the last, and contended for it strenuously and courageously, but instead of adherents flocking to his standard he had the discouragement of seeing their number grow annually less; while every new fact brought to light in the science of life, and every new work published on the subject, went to swell the tide against which he was struggling. It was to no purpose that he issued tome after tome of ponderous learning. The evil was not checked—the current grew continually stronger, and he has at last concluded to let it take its course. Dr. Paine is the last of his peculiar race of physiologists. Henceforth no voice, in any of our schools, will be raised in favor of "Solidism" against "Vital Chemistry," now that his is to be heard on that question no more. To his chemical colleague physiology is confided, to be taught according to the strictest sect of the chemists. Dr. Draper may make it too chemical, but he will not fail to render it highly interesting to his pupils. He is an original thinker, and his speculations and experiments have attracted much attention. This new adjustment of duties in the New York University is unquestionably judicious.

It was announced, some months ago, that Dr. Drake had resigned his professorship in the Medical College of Ohio, but we supposed that his resignation would not be accepted, and that an arrangement would be made by which his connection with the school would be continued. We could not believe that he was about to part so summarily with an institution whose image had haunted and delighted him so long. If it drew him away from Lexington and Philadelphia, and interposed between him and a class of "four hundred and six pupils in Louisville," and rendered him blind to fair prospects and deaf to pleasant voices every where else, we could not pursuade ourselves that he would abandon it after one short winter. But such seems to be the fact; Dr. John Bell is announced as his successor in the Medical Examiner for July. Dr. Drake is a teacher of great experience, and of varied and extraordinary powers, and his resignation would be felt as a loss by any institution in our country; but the Medical College of Ohio is fortunate in having secured the services of a gentleman worthy to succeed him in the Chair of the Theory and Practice of Medicine. Dr. Bell is known to every medical reader in America as an author of high repute, and his standing at home and among his professional brethren, as a high-toned gentleman, is as enviable as the reputation he has won, as an author. We give him a hearty welcome to our side of the mountains, and shall rejoice if the future success of the school to which he has united himself prove equal to his expectations and deserts.

We have understood that Dr. Bayless has also resigned his Chair in the Medical College of Ohio, and that Dr. Shotwell returns to it, though we have not seen an official statement of the fact.

The Lexington Medical School has, at length, gone out of existence; Dr. Dudley resigned his place in it a short time since. No circular has been issued, and it is understood that none will be issued, announcing lectures there for the coming winter. There is something melancholy in the demise of this, the oldest Medical School in the West, although the event is one which, for some time past, was clearly foreseen as inevitable. If there had been nothing in its site fatal to its continuance, there had been of late years that in its administration which must have tried to the utmost its powers of endurance. No institution in the world could resist the force of both combined; and Transylvania has gone down, not with a crash, startling the profession, but wasted by a slow decay. Her last class numbered, as her professors declared to the board of trustees, some fifty odd students. It was time that a school, long first in the Valley of the Mississippi, had closed her doors when she had come to this. We suppose Dr. Dudley, ere this, has become convinced that it is too late in the day to attempt to sustain a Medical School without a hospital and without subjects for dissection.

Professor Wood is transferred to the Chair of Theory and Practice in the University of Pennsylvania, made vacant by the resignation of Dr. Chapman, and Dr. Joseph Carson has been elected Professor of Materia Medica ond Pharmacy. Y.

